# An open source and convenient method for the wide-spread testing of COVID-19 using deep throat sputum samples

**DOI:** 10.7717/peerj.13277

**Published:** 2022-05-10

**Authors:** Sunny C. Huang, Thomas K. Pak, Cameron P. Graber, Charles C. Searby, Guanghao Liu, Jennifer Marcy, Alexandra K. Yaszemski, Kurt Bedell, Emily Bui, Stanley Perlman, Qihong Zhang, Kai Wang, Val C. Sheffield, Calvin S. Carter

**Affiliations:** 1 Medical Scientist Training Program / Roy J. and Lucille A. Carver College of Medicine, University of Iowa, Iowa City, IA, United States of America; 2 Stead Family Department of Pediatrics/ Division of Medical Genetics and Genomic / Roy J. and Lucille A. Carver College of Medicine, University of Iowa, Iowa City, IA, United States of America; 3 Department of Neurology, University of Iowa Hospitals and Clinics, Iowa City, IA, United States of America; 4 Department of Neurology / Mayo Clinic Graduate School of Biomedical Sciences, Mayo Clinic, Rochester, MN, United States of America; 5 Department of Microbiology and Immunology / Roy J. and Lucille A. Carver College of Medicine, University of Iowa, Iowa City, IA, United States of America; 6 Department of Biostatistics / University of Iowa College of Public Health, University of Iowa, Iowa City, IA, United States of America; 7 Department of Ophthalmology and Visual Sciences, University of Iowa Hospitals and Clinics, Iowa City, IA, United States of America

**Keywords:** COVID-19, Saliva, SARS-CoV2, Pooling, Virus testing, Gene pool

## Abstract

**Importance:**

The rise of novel, more infectious SARS-CoV-2 variants has made clear the need to rapidly deploy large-scale testing for COVID-19 to protect public health. However, testing remains limited due to shortages of personal protective equipment (PPE), naso- and oropharyngeal swabs, and healthcare workers. Simple test methods are needed to enhance COVID-19 screening. Here, we describe a simple, and inexpensive spit-test for COVID-19 screening called Patient Self-Collection of Sample-CoV2 (*PSCS-CoV2*).

**Objective:**

To evaluate an affordable and convenient test for COVID-19.

**Methods:**

The collection method relies on deep throat sputum (DTS) self-collected by the subject without the use of swabs, and was hence termed the Self-Collection of Sample for SARS-CoV-2 (abbreviated *PSCS-CoV2*). We used a phenol-chloroform extraction method for the viral RNA. We then tested for SARS-CoV-2 using real-time reverse transcription polymerase chain reaction with primers against at least two coding regions of the viral nucleocapsid protein (N1 and N2 or E) of SARS-CoV-2. We evaluted the sensitivity and specificity of our protocol. In addition we assess the limit of detection, and efficacy of our Viral Inactivating Solution. We also evaluated our protocol, and pooling strategy from volunteers on a local college campus.

**Results:**

We show that the *PSCS-CoV2* method accurately identified 42 confirmed COVID-19 positives, which were confirmed through the nasopharyngeal swabbing method of an FDA approved testing facility. For samples negative for COVID-19, we show that the cycle threshold for N1, N2, and RP are similar between the *PSCS-CoV2* and nasopharynx swab collection method (*n* = 30). We found a sensitivity of 100% (95% Confidence Interval [CI], 92-100) and specifity of 100% (95% CI, 89-100) for our* PSCS-CoV2* method. We determined our protocol has a limit of detection of 1/10,000 for DTS from a COVID-19 patient. In addition, we show field data of the *PSCS-CoV2* method on a college campus. Ten of the twelve volunteers (N1 < 30) that we tested as positive were subsequently tested positive by an independent laboratory. Finally, we show proof of concept of a pooling strategy to test for COVID-19, and recommend pool sizes of four if the positivity rate is less than 15%.

**Conclusion and Relevance:**

We developed a DTS-based protocol for COVID-19 testing with high sensitivity and specificity. This protocol can be used by non-debilitated adults without the assistance of another adult, or by non-debilitated children with the assistance of a parent or guardian. We also discuss pooling strategies based on estimated positivity rates to help conserve resources, time, and increase throughput. The *PSCS-CoV2* method can be a key component of community-wide efforts to slow the spread of COVID-19.

## Introduction

The SARS-CoV-2 is an infectious coronavirus, which can lead to acute respiratory distress syndrome (ARDS) in some patients. The disease was first identified in Wuhan, China in 2019 and hence called Corona Virus Disease-2019 (COVID-19) (World Health Organization, 2020). The virus itself was provisionally named 2019-nCoV and later changed to severe acute respiratory syndrome coronavirus 2 (abbreviated SARS-CoV-2) (Gorbalenya et al., 2020). Since the time the disease was first detected in December 2019, it has spread across the world resulting in the current pandemic. The majority of cases result in mild symptoms such as fever, cough, sore throat, fatigue, loss of taste and/or smell, muscle pain, abdominal pain, headache, but severe cases may progress to pneumonia, multi-organ failure and death (Zhu et al., 2020; Huang et al., 2020; Wang et al., 2020).

The COVID-19 virus, SARS-CoV-2, has affected nearly 200 million people and was responsible for 4.2 million deaths worldwide (World Health Organization, 2021). These numbers are likely an underestimate and will continue to grow despite the rollout of vaccines. Concerningly, a more infectious SARS-CoV-2 variant, B.1.617.2 (Delta), has arisen. The delta variant was first detected in India in December 2020 and was responsible for the deadly second wave of COVID-19 cases in India in April 2021 (Aleem, Samad & Slenker, 2021). The rapid global spread of the delta variant prompted the World Health Organization (WHO) to classify it as a variant of concern in May 2021 (Aleem, Samad & Slenker, 2021). In the United States, the delta variant accounted for 83% of the COVID-19 cases in July 2021 (Centers for Disease Control and Prevention, 2021). Convenient and accessible testing of SARS-CoV-2 was essential in controlling the COVID-19 pandemic.

The ability to efficiently test for COVID-19 in potentially exposed individuals and communities is beneficial in reducing the spread of the virus, especially in developing nations where vaccination is limited. In addition, widespread testing facilitates scientific studies to better understand the disease epidemiology (Lipsitch, Swerdlow & Finelli, 2020). Countries that applied generalized testing have been reported to have better contained COVID-19 (Normile, 2020; Bennhold, 2020). However, in developing countries, limitations on wide-spread testing were still present due to various obstacles such as poorly organized efforts (Maxmen, 2020), a shortage of nasopharyngeal/oropharyngeal swabs and the chemicals needed to run the test, the lack of availability of personal protective equipment (PPE), and the logistic of having enough healthcare workers wearing approved PPE for the physical collection of specimens from patients using a process that may expose healthcare workers to the virus and was uncomfortable, or even painful to subjects.

To overcome these barriers, a testing method should allow individuals to self-collect easily obtainable bodily fluids such as saliva and sputum samples without the physical assistance of, or close contact with a healthcare worker. In addition, employing a method that relies on inexpensive and readily available reagents to extract the RNA from these samples would greatly facilitate the widespread testing for COVID-19 around the globe. Here we describe a method termed Patient Self-Collection of Sample for SARS-CoV-2 (hereafter abbreviated as *PSCS-CoV2*) and the acid guanidinium thiocyanate-phenol-chloroform (AGPC) method of RNA extraction to solve this problem. *PSCS-CoV2* is a deep throat sputum (DTS) fluid collection protocol and specimen kit, which allows patients to self-collect samples. A review paper noted DTS as “sputum sampling is performed by throat clearing and coughing up and out the secretion and sputum of the retropharynx” (Won et al., 2020). DTS contains both sputum and saliva. While saliva can be accurate for SARS-CoV-2 detection (Won et al., 2020), we collected sputum to cover more of the respiratory tract, since COVID-19 is a respiratory infection. In addition, the review paper noted better accuracy with DTS that required coughing (Won et al., 2020), which we require in our protocol. The Two-Step Kit container allows for the inactivation of virus within the container once the patient has self-collected the sample. The sample was then processed in a certified laboratory in a BSL2+ hood to isolate the viral RNA for detection using real-time RT-PCR testing to detect nucleic acid from the SARS-CoV-2. The AGPC has recently been found to be suitable for SARS-CoV-2 PCR detection (Khiabani & Amirzade-Iranaq, 2021). The AGPC was a simple method used in many research institutions and laboratories worldwide. The method itself has a long-known track record, and commercially mixed reagents needed for this step are readily available from several vendors at a low cost (Chomczynski & Sacchi, 1987). Dimke et al. has shown that AGPC can be used as an alternative to automated systems for the RT-qPCR detection of SARS-CoV-2 (Dimke et al., 2021). They noted the key advantages of the AGPC is scalability and low costs (Dimke et al., 2021).

The purpose of this paper is three-fold: (1) present an open-source method by which individuals can self-collect samples for COVID-19 molecular testing; (2) demonstrate the accuracy and sensitivity of the collection method; and (3) proof of concept of an algorithm for pooling patient samples.

## Materials & Methods

### Informed consent

All study participants were enrolled and sampled in accordance to the University of Iowa IRB-01 approved protocol #: 202004568. All study participants gave consent, but we had a waiver of documentation of consent. Recruitment was conducted through advertisements, word of mouth, and referral from a physician caring for COVID-19 patients, with the patients agreeing for us to contact them by email or telephone. Clinical data and samples were only gathered after the study participant had acknowledged that they understood the study protocol and consented. All participant information and samples were gathered in association with study identifiers. After data analysis, we destroyed the link between the ID code and subject identifiers.

### Collection of positive and negative samples from the community

The study was conducted prospectively. We collected samples from patients that were deemed positive or negative for SARS-CoV2. We aimed to collect a minimum of 30 positive samples and 30 negative samples in order to ensure that our 95% confidence interval for 30/30 would have a minimal of 89% for specificity and sensitivity.

For positive cases, we obtained samples from patients presenting with signs and symptoms of COVID-19, who underwent NP swab collection of specimens at the University of Iowa Hospitals and Clinics (UIHC). These samples were processed and tested using the CDC approved methods (EUA2000001). Subsequently, patients who tested positive at an FDA approved testing center were recruited and enrolled into our IRB-approved study after obtaining consent. Following enrollment, patients independently self-collected DTS samples. Samples were collected by the patient within 72 h of being tested positive at an FDA approved testing center. It should be noted that most samples were collected within 24 h of testing positive at an approved testing center. All samples were transported at ambient temperatures, and tested within 48 h of collection using the PSCS-SARS-CoV2 test. **A positive in the** ***PSCS-CoV2*** **assay was determined to be a cycle threshold < 40 for N1 and < 40 for a second SARS-CoV-2 marker, N2 or E**. This threshold was set from earlier recommendations from CDC. An RP of < 34 was needed to ensure the integrity of the sample.

Negative cases were recruited based on the absence of symptoms including absence of a history of a fever for the past month and lack of contact with known positive patients. The subjects were provided instructions and independently self-collected DTS fluid. Nasopharyngeal swabs from the subjects were also collected by a trained medical provider. All samples were transported at ambient temperatures and tested within 48 h of collection using the PSCS-SARS-CoV2 test, unless noted in the results. A negative sample was determined to be a cycle threshold > 40 for N1 and > 40 for a second SARS-CoV-2 marker, N2.

### Collection of field samples

Field samples were recruited from a local college campus. A positive was determined to be a cycle threshold < 40 for N1 and < 40 for a second SARS-CoV-2 marker N2 or E. Samples that did not reach positive criteria for both markers were scored as negative. Samples that were positive for one marker and negative for the other marker were scored as ambiguous and retested. Participants were notified of their result and asked to self-report their COVID results from an FDA approved testing site. For the samples used for the pooling strategy, individual samples were tested if they had a cycle threshold < 40 for N1 or < 40 for a second SARS-CoV-2 marker (N2 or E1). We used a different second SARS-CoV-2 marker, E1, in some cases to test the validity of our original N2 SARS-CoV-2 marker.

### Patient self-collection of sample for SARS-CoV-2

*PSCS-CoV2* is a deep throat sputum (DTS) fluid collection protocol and specimen kit, which allows patients to self-collect a sample without the physical assistance of or close contact with another individual, most importantly without the assistance of a healthcare worker. The fluid collected contains both sputum and saliva. The Two-Step Kit container allows for the inactivation of virus within the container once the patient has self-collected the sample. The containers were placed in a biohazard bag, and handled with caution. Samples were then processed in a certified laboratory in a BSL2+ hood to isolate the viral RNA for detection using real-time RT-PCR testing intended for the presumptive qualitative detection of nucleic acid from the SARS-CoV-2. We obtained samples from individuals diagnosed with COVID-19, or individuals negative for COVID-19, or individuals with signs and symptoms of infection who are suspected of COVID-19 or individuals exposed to COVID-19, such as healthcare workers, family members of COVID-19 patients, individuals living in care facilities, etc.

### Materials

#### Collection and virus inactivating containers

Here, we present a Two-Step Kit for sample collection, which consists of a sample collection tube (a 50 mL self-standing centrifuge tube with cap; #430921; Corning, Inc., Corning, NY, USA) and a smaller tube (5 mL self-standing tube with cap; VWR #89497-728). The smaller tube contains a virus-inactivating and RNase-inhibiting solution (abbreviated VIS), and the 5 mL tube was hereafter referred to as the Virus Inactivating Solution (VIS) container. Picture of the sample collection kit is shown in Fig. S1.

#### Virus inactivating and RNase inhibiting solution (VIS)

VIS consists of the combination of guanidine thiocyanate (4 M), sodium citrate (25 mM at pH 7.0) and N-laurosylsarcosine (0.5% wt/vol).

#### Preparation of two-step kit

Under sterile conditions, 1.5 mL of VIS was placed in a sterile RNase and DNase-free VIS container. The VIS container was capped and placed inside of a 50 ml collection container. The larger 50 mL container plus the smaller container holding 1.5 mL of VIS was placed inside of a biohazard bag with instructions specific for the Two-Step Kit.

### *PSCS-CoV2* two-step protocol

1. Do not eat, drink, smoke, or chew gum for 30 min prior to use.
2. The subject was provided a Two-Step Kit, which contains instructions, one sealable biohazard plastic bag, one large sample collection tube, one VIS container, and a prelabeled sticker with the patient’s ID information to identify the sample.
3. The subject opens the kit and places the ID sticker on the side of the large sample collection container. The subject then removes the cap from the large collection container and sets the small VIS container to the side for later use in step 5.
4. The subject, while isolated from other individuals, snorts through the nose with mouth closed by taking a deep breath inward, then coughs to clear the throat attempting to bring up phlegm (phlegm was NOT a requirement for a good sample), and spits into the large collection container. Only a small volume was needed (1 spit was usually plenty, usually yielding ∼0.5 mL).
5. The subject then takes the VIS container, removes the lid, and pours the liquid into the large collection container. The subject throws away the small VIS container.
6. The subject caps the large collection container tightly and shakes vigorously for ∼30 s to mix the collected sample with VIS.
7. Subject places the container back in the provided sealable biohazard plastic bag. The sample was now ready for pick-up or drop-off.

**Subsequent testing for SARS-CoV2 in self-collected samples obtained by the** ***PSCS-CoV2*** **method**: We use the classic single step phenol-chloroform RNA preparation protocol (Chomczynski & Sacchi, 2006), with some minor modifications, beginning with 500 µL of the collected sample (mixed with VIS). Once the RNA was isolated and was in dry pellet form, the pellet was resuspended in 20 µL of RNase-free water, 2.5 µL of the resuspended RNA was used for real-time RT-PCR. The remaining sample may be stored for replications, as needed.

### Real-time RT-PCR

The specific nucleic acid sequences from the genome of SARS-CoV-2 [2019-nCoV_PH_nCOV_20_026 N gene for nucleocapsid phosphoprotein] are used for RT-PCR primers. Specifically, primers for real-time RT-PCR for N1, N2 and E sequences are used. These primers are provided by Integrated DNA Technologies (IDT) (Iowa City, IA, USA) in their 2019-nCoV CDC EUA Kit (IDT Catalog #10006606). This kit contains primers against two regions of the RNA sequence for the nucleocapsid phosphoprotein (referred to as N1 and N2), envelope protein (E) and the human RNase Protein (RP) as a positive collection control. We initially used a cDNA template control for the N1 and N2 sequences. However, early in the study, we were also able to acquire an RNA control containing the SARS-CoV-2 genome from a COVID-19 positive patient. We used this sample as a positive control. Sequencing of the RNA control confirmed that the sample was SARS-CoV-2.

The N1 and N2 are commonly used to test for SARS-CoV-2 with no concerns about alteration of analytical sensitivity characteristics of viral detection for the delta variant. The delta variant is not mutated in the N1, N2, or E primer or probe sequences.

Real-time RT-PCR was performed using the protocols as described in the CDC 2019-Novel Coronavirus (2019-nCov) Real-Time RT-PCR Diagnostic Panel (Catalog#2019-nCoVEUA-01; effective 3/30/2020) (Ref. 10), with the exception that a Bio-Rad CFX96 Real-Time PCR instrument was used, rather than the Applied Biosystems 7500 Real-time PCR instrument.

### Viral inactivating solution assessment study

We used mouse hepatitis virus (MHV), strain A59, a beta coronavirus that is closely related to SARS-CoV-2. Fifty microliters of VIS, VIS-DTS (1:3), or PBS were mixed 1:1 with 50 µL MHV at varying concentration of viral titers and incubated for 10 min at ambient temperature. Live virus titers were determined using a plaque assay of HeLa cells expressing the MHV receptors as described in a previous paper (Grunewald et al., 2020).

### Limit of detection study

A DTS sample was collected from a COVID-19 patient previously confirmed positive by an independent Clinical Laboratory Improvement Amendments (CLIA) approved lab. We isolated the RNA from this sample using the described phenol/chloroform extraction method. The RNA from this sample was diluted with DTS from a known negative subject at various dilutions to determine the limit of detection (LoD). The LoD is the greatest dilution of a DTS sample from a COVID-19 patient to still test as positive with our protocol. The various dilutions we tested were 1/10, 1/100, 1/10^3^, 1/10^4^ and 1/10^5^. The positive COVID-19 DTS sample was diluted in a known negative DTS sample. The RNA was extracted using the phenol/chloroform method and assayed using our real-time RT-PCR method (PSCS-SARS-CoV2). These samples were then assayed in triplicate, and we determined that 1/10^4^ was the LoD.

The 1/10^4^ dilution was then used to spike 20 aliquots of DTS at 2xLoD (1/5000 final dilution) from known negative subjects. These spiked samples were processed with the phenol/chloroform extraction method and assayed using the downstream assay (PSCS-SARS-CoV2) with primers and probes for N1, E and RP.

### Data analysis

Data analysts and experimenters were blinded to the COVID status of the samples. The investigators collecting the sample were aware of the COVID status and unblinded the results after data analysis. Cycle threshold for determining the sensitivity and specificity of the samples were determined prospectively. The Wilson/Brown method was used to calculate the 95% confidence interval for sensitivity and specificity. The cycle threshold for the field data and pooling data were determined retrospectively, after the data were collected. Statistical analysis and graphing was performed using GraphPad Prism 8.2.0.

## Results

### Clinical validation of SARS-CoV-2 using the patient self-collection of DTS method

To test the accuracy of the Patient Self-Collection of Sample-CoV2 (*PSCS-CoV2*), we recruited COVID-19 positive and negative patients. A total of 42 positives patients (confirmed positive from an independent CLIA approved laboratory) and 30 negative subjects (confirmed negative with nasopharyngeal swab samples) underwent sample collection using the *PSCS-CoV2* collection kit as described previously in this paper. The DTS samples were tested using real-time RT-PCR as described in the methods. The experimenter and data analysts were blinded to the COVID status of the samples. There was a 100% agreement between the results (*i.e.,* positive or negative) obtained from testing of self-collected samples and those obtained from nasopharyngeal swabs (Table 1). Hence, our method had 100% specificity (95% CI [89–100]) and 100% sensitivity (95% CI [92–100]). We show a more detailed assessment of the SARS-CoV2 positive and negative samples in Table S1.

We also serially obtained samples using the *PSCS-CoV2* collection method from a COVID-19 positive individual. The patient remained positive for at least 10 days after the initial positive test (Fig. S2). Notably, the DTS samples show reduced copies of viral RNA (increasing cycle threshold) over time.

### Limits of detection experiments using SARS-CoV2 RNA

The Limit of Detection (LoD) study established the lowest SARS-CoV2 viral concentration detectable on DTS collected using the *PSCS-CoV2* method. LoD was determined in two phases. First, positive SARS-CoV2 RNA at concentrations of 1/10, 1/100, 1/10^3^, 1/10^4^ and 1/10^5^ were assayed in triplicate. Results of this experiment show that viral RNA for all dilutions were positive in triplicate (Table 2). Second, we selected the 1/10^4^ dilution as the preliminary LoD because the cycle threshold (Ct) was lower than 30 for all three markers, indicating higher stringency than 1/10^5^ dilution. The 1/10^4^ dilution was confirmed with 20 contrived replicates at final 1/5000 dilution (2xLoD), showing 100% detection of SARS-CoV2 as shown by Ct < 40 for N1 and E (Table 2).

Overall, the *PSCS-CoV2* method has a limit of detection of at least a 1/10^4^ dilution of a DTS sample from a positive patient. We estimate that there are approximately 100 copies of the viral RNA in a 1/10^4^ dilution. This was based on comparison of a SARS-CoV2 viral RNA sample with a quantified viral cDNA (plasmid) sample using quantitative PCR. A plasmid positive control sample of 100 copies gave an approximate Ct of 25, similar to the Ct of the 1/10^4^ viral RNA dilution.

**Table 1:** Confusion matrix of PSCS-CoV2 for detection of SARS-CoV2 positive and negative samples.

		**SARS-CoV2 status (nasopharyngeal swab)**	
		**Positive**	**Negative**
**PSCS-CoV2** **(Sputum)**	**Positive**	**42**	**0**
	**Negative**	**0**	**30**

**Notes.**

PSCS-CoV2 = method of patient self-collection of sample for SARS-CoV-2.

**Table 2:** Limit of detection determination. There was 100% detection of SARS COV-2 RNA at dilutions of 1/10, 1/100, 1/10^3^, 1/10^4^, 1/10^5^, and 1/5000.

**Dilution of positive sample**	**Replicate**	**Ct value**			**Positive/Total**
		**N1**	**E**	**RP**	
1	**Mean Ct (SD)**	13.2 (0.2)	15.9 (0.1)	21.8 (0.1)	3/3
1/10	**Mean Ct (SD)**	15 (0.9)	18.1 (1.2)	28.1 (0.2)	3/3
1/100	**Mean Ct (SD)**	17.7 (0.2)	21.2 (0.3)	24.2 (0.2)	3/3
1/10∧3	**Mean Ct (SD)**	21.4 (0.5)	24.7 (0.8)	25 (0.3)	3/3
1/10∧4	**Mean Ct (SD)**	24.5 (0.3)	27.9 (0.3)	24.7 (0.3)	3/3
1/10∧5	**Mean Ct (SD)**	28.1 (0.3)	31.1 (0.4)	24.4 (0.3)	3/3
**1/5000 (2X LoD)**	**Mean Ct (SD)**	24.2 (0.9)	27.1 (0.7)	25.6 (0.5)	20/20

**Notes.**

Ct cycle threshold SD standard deviation

### Assessment of virus inactivation solution

To ensure that the Viral Inactivating Solution does not affect the accuracy of our *PSCS-CoV2* method, we performed an analytical equivalency study. We tested the samples collected in our specific self-collection container (containing virus inactivating reagents) *versus* those collected in conventional collection containers (without any virus inactivating regents). To test COVID-19 positive samples, we used 1/5000 of a DTS from a positive COVID-19 patient 92x LoD). A total of 10 replicates for COVID-19 positive samples and a total of 10 replicates for COVID-19 negative samples (Table 3). There was 100% agreement between the two containers (with and without VIS). 100% of the spiked samples were positive for SARS-CoV-2 (with and without VIS), and 100% of the non-spiked samples were negative (with and without VIS). Of note, the Ct values were not significantly different between No VIS and VIS samples (*p* > 0.05) for any of the markers (N1, E, and RP).

**Table 3:** Viral inactivation solution analytical equivalency study. There was a 100% agreement with samples positive for SARS-CoV2 (with and without VIS) and a 100% agreement with sample negative for SARS-CoV2 (with and without VIS).

***Concentration of positive SARS-CoV2 RNA in saliva***	***Replicates***	***No VIS: Mean Ct (SD)***			***VIS Ct: Mean Ct (SD)***			***% Agreement***
		***N1***	***E***	***RP***	***N1***	***E***	***RP***	
**2x LoD**	10	28.3 (1.3)	28.8 (1.3)	22.6 (0.3)	26.4 (3.3)	27.8 (2.2)	23.3 (0.5)	100%
**Negative (no viral RNA added)**	10	*n.d.*	*n.d.*	22.5 (.2)	*n.d.*	*n.d.*	23.6 (0.4)	100%

**Notes.**

n.d. = not detected, cycle threshold growth curves do not cross the baseline before 40 cycles.

SD Standard deviation VIS Viral inactivating solution

To confirm inactivation of the infectious virus with VIS, we used the plaque assay method using mouse hepatitis virus (MHV), strain A59, a beta coronavirus that is closely related to SARS-CoV-2. The plaque forming unit (PFU) is a measure of the live virus titer. Following our protocol methods, treatment with VIS alone reduced live virus titers by approximately 99.9% and with VIS/DTS by at least 99.9% (PBS control: 2.2 × 106 PFU/mL; VIS: 3.75 × 103 PFU/mL; VIS/DTS: < 1.25 × 103 PFU/mL) (Calculations are in the Raw Data file).

### RNA stability using the *PSCS-CoV2* method with viral inactivating solution

We performed RNA specimen stability experiments using spiked samples in Viral Inactivation Solution (VIS). Testing RNA stability was critical since DTS samples may be subject to extreme temperatures on transit to the diagnostic laboratory. So we incubated the DTS samples at various temperatures, and for a duration of time that samples could be in transit. We had three groups of samples, samples spiked with 10x LoD for SARS-CoV2 RNA, samples spiked with 2x LoD for SARS-CoV2 RNA, and samples negative for SARS-CoV2 samples. We tested the three groups at 40 °C for 56 h (Table 4), 25 °C for 56 h (Table 5), and 4 °C for 56 h (Table 6). There was a 100% agreements for all three groups in detecting N1, E, and RP.

### Field data of the *PSCS-CoV2* method from a college campus

We tested subjects from a college campus for SARS-CoV-2 during the Fall 2020 . A subject was deemed positive for SARS-CoV-2 if their sample had a cycle threshold < 40 for N1 and < 40 for a second SARS-CoV-2 marker, N2. Of the 306 subjects tested by our *PSCS-CoV2* method, 10.4% tested positive.

To provide an approximation of the accuracy of our *PSCS-CoV2* method, we collected the results from subjects who were subsequently retested at an independent CLIA approved laboratory. The subjects underwent a nasopharygeal swab within a week of their initial *PSCS-CoV2* test. Release of the results to us was entirely voluntary by the subjects and 23 subjects volunteered (Table 7). Ten of twelve (10/12) samples with an N1 < 30 by our test were confirmed positive. One of eight (1/8) samples with an N1 > 30 were confirmed positive. In addition, three of three (3/3) samples that were tested negative, were confirmed negative.

**Table 4:** Summary of sample stability at 40 °C for 56 h.

**Concentration**	**Analysis**	**Mean Ct**		
		**N1**	**E**	**RP**
**10x LoD**	Positives/Total	10/10	10/10	10/10
	Mean Ct ± SD	17.4 ± 0.3	21.1 ± 0.3	24.5 ± 0.4
**2x LoD**	Positives/Total	20/20	20/20	20/20
	Mean Ct ± SD	20.5 ± 0.4	23.7 ± 0.4	26.0 ± 0.2
**Negative**	Positives/Total	0/10	0/10	10/10
	Mean Ct ± SD	n.d.	n.d.	20.1 ± 0.7

**Notes.**

n.d. = not detected, cycle threshold growth curves do not cross the baseline before 40 cycles.

SD Standard deviation LoD Limit of detection

**Table 5:** Summary of sample stability at 25 °C for 56 h.

**Concentration**	**Analysis**	**Mean Ct**		
		**N1**	**E**	**RP**
**10x LoD**	Positives/Total	10/10	10/10	10/10
	Mean Ct ± SD	19.7 ± 0.2	20.7 ± 0.2	23.2 ± 0.3
**2x LoD**	Positives/Total	20/20	20/20	20/20
	Mean Ct ± SD	22.9 ± 0.7	23.5 ± 0.5	24.1 ± 0.2
**Negative**	Positives/Total	0/10	0/10	10/10
	Mean Ct ± SD	n.d.	n.d.	20.9 ± 1.2

**Notes.**

n.d. = not detected, cycle threshold growth curves do not cross the baseline before 40 cycles.

SD Standard deviation LoD Limit of detection

**Table 6:** Summary of sample stability at 4 °C for 56 h.

**Concentration**	**Analysis**	**Mean Ct**		
		**N1**	**E**	**RP**
**10x LoD**	Positives/Total	10/10	10/10	10/10
	Mean Ct ± SD	20.4 ± 0.2	21.1 ± 0.2	23.9 ± 0.3
**2x LoD**	Positives/Total	20/20	20/20	20/20
	Mean Ct ± SD	23.4 ± 0.7	24.0 ± 0.5	24.6 ± 0.3
**Negative**	Positives/Total	0/10	0/10	10/10
	Mean Ct ± SD	n.d.	n.d.	22.8 ± 1.7

**Notes.**

n.d. = not detected, cycle threshold growth curves do not cross the baseline before 40 cycles.

SD Standard deviation LoD Limit of detection

**Table 7:** Secondary testing of field samples for SARS-CoV-2. For our *PSCS-CoV2* method, a positive was a sample with N1 < 40 and a N2 < 40. Samples that did not meet that threshold were deemed negative. Retests were performed by an independent Clinical Laboratory Improvement Amendments (CLIA) approved laboratory using nasopharyngeal swabs for sample collection.

**Category**	**Initially tested positive (PSCS-CoV2)**	**Re-tested positive**	**Percent agreement**
N1 < 30	12	10	83.3
N1 > 30	8	1	12.5
**Category**	**Initially tested negative (PSCS-CoV2)**	**Re-tested negative**	**Percent agreement**
N1 > 30	3	3	100

We hypothesized that the majority of the samples with an N1>30 would retest as negative since the subjects would be at the tail end of the disease. With the virus titer already low, it would be even lower by the time the subjects were retested. The sample that was retested as positive with an N1 > 30 were likely due to the patients being at the beginning stages of their infection. This test was also subject to response bias since those likely to get retested were those who were noted as positive for COVID-19.

### Pooling strategy

To increase the time and resource efficiency for COVID-19 testing, we employed a pooling strategy. First, we combined equal aliquots of samples into a new tube. Second, the pooled sample was then tested for SARS-CoV-2. Third, from the pools tested positive for SARS-CoV-2, indivdual samples were tested for SARS-CoV-2.

We mathematically developed a pooling strategy for pool size based on positivity rate. Let k be the pool size and p the SARS-CoV-2 positivity rate. With probability (1 − *p*)^*k*^, the pool is negative. In this case one test was enough. Otherwise *k* +1 tests are needed (1 for the pool followed by k individual tests). The expected number of tests with pooling was (1 − *p*)^*k*^+ [1 − (1 − *p*)^*k*^](*k* + 1). Pooling was meaningful only when this number was smaller than *k*, the number of individual tests. This implies *p* < 1 − (1/*k*)^1/*k*^. **For pool size** ***k*=2,4,6, and 8, p needs to be less than 0.292, 0.292, 0.258, and 0.229, respectively**. Interestingly, for *k* = 2 and 4, the thresholds are the same.

We also developed the following equation to describe the max number of tests one would have to perform in the worst case scenario. The assumption was that each positive sample ends up in its own pool. We based the equation on the same COVID-19 positivity rate (*p*), pool size (*k*) and total number of individual samples (*s*).

Max number of trials = (*s/k*) + (p*s*k).

For a more conservative approach in determining the pool size based on the *p*, we utilized this equation of “max number of trials”. To ensure that the “number of combined trials in the pooling method” was never more than the standard method of “testing every individual sample”, the equation would be the following: *s* < (*s*/*k*) + (*p*∗*s*∗*k*), or 1 < 1/*k* + (*k*∗*p*). The requirement for this equations is that *s* > 2*k* because there need to be enough samples to make multiple pools. In addition, the assumption is that $p\lt \frac{1}{k} $ since a large positivity rate would void the equation. **For pool size** ***k*=2, 4, 6, and 8,** ***p*** **needs to be less than 0.250,0.188,0.139,0.109,** respectively.

We explored pooling stategy of pools of four subjects (Pool A) and pools of two subjects (Pool B) noted in Table 8. Individual samples were tested in a pool that had a cycle threshold <40 for N1 or <40 for a second SARS-CoV-2 marker (E). In our on-field data of a college campus, we had 12 pools of A (pool size of four, average CT of RP was 20.9). Three pools of A were positive. Altogether, we identified four individual positive samples (8.3%). Therefore, instead of having to test 48 individual samples, we only had to test 24 samples (12 pools and 12 individual samples) to identify the four COVID-19 positive patients.

For Pool B, we had 14 pools (pool size of 2, average CT of RP was 20.7). Three Pools of B were were positive. Altogether, we identified four individual positive samples (13%). So instead of having to test 28 individual samples, we only had to test 20 individual sample (14 pool and six inidividual samples) to identify the four COVID-19 positive patients.

## Discussion

The existence of recent variants has demonstrated the importance of continued COVID-19 testing to control the COVID-19 pandemic. Fortunately, the primers for detection of SARS-COV2 remains unchanged for the delta and omicron variants. Substantial bottlenecks have limited the capacity for widespread COVID-19 testing. PPE, swabs, assay reagents and healthcare workers are all vital to achieve nationwide testing but have been in short supply. There are at least three ways to solve a supply shortage: increased production, reduced utilization, and innovation. Here, we demonstrate the validity and sensitivity of a DTS-based collection protocol that is both innovative in design and at the same time reduces the reliance on resources in short supply over the traditional testing method.

**Table 8:** Results of pooling strategy.

Pool type	SARS-CoV2 positivity rate (p)	Size of the pools (k)	Total number of individual samples (s)	Number of individual samples positive for SARS-COV-2	Total number of tests	Number of tests saved by the pooling method
Pool A	0.083	4	48	4	24	24
Pool B	0.13	2	28	4	20	8

**Notes.**

Pool A = pools of four individual subjects. Pool B = pools of two individual subjects. Total Number of Tests = (number of pools + number of individual samples tested).

The *PSCS-CoV2* sample self-collection protocol has at least 10 key advantages over currently utilized swab-based collection protocols: 1. The protocol does not use nasopharyngeal or oropharyngeal swabs; 2. The protocol allows most patients to collect the sample themselves (self-collection) and thus, the protocol does not expose a healthcare worker to pathogens during the collection process; 3. Because healthcare workers are not needed to collect the sample from the patient/subject, personal protective equipment (PPE) is not required for the collection process, thus conserving PPE; 4. There is no need for close contact with medical or technical staff, until the sample has been partially processed to inactivate the infectious agent; 5. The current protocol does not utilize RNA processing kits or qPCR reagents; 6. Abundant and high-quality RNA can be readily isolated from the collected sample and is comparable in nature to that obtained by using swabs; 7. The collection container is a simple conical tube to which the viral inactivating agent is added; 8. The *PSCS-CoV2* protocol is suitable for offsite (out of hospital or clinic) high-throughput collection of patient samples, such as “drive through testing”; 9. Since the viral inactivating agent also preserves RNA, the sample does not need to be processed immediately; and 10. The ease and non-invasive means of the *PSCS-CoV2* self-collection can help with adherence in the context of serial collection of samples for research, or regular testing programs.

One of the main advantages of our method is self-collection. While self-collection using swabs may be possible for some, it is not generally feasible or desirable for many. The swabs are physically uncomfortable, and there is a small risk for nasal bleeds or the swab breaking (Koskinen et al., 2021). Yet, most tests currently available rely on the use of nasopharyngeal or oropharyngeal swabs to obtain samples (Webber & Jewett, 2021). Our experiments comparing nasopharnygeal swab *versus PSCS-CoV2* DTS collection indicated that the use of swabs is not necessary. There are special conditions where swabs may be useful, such as for debilitated patients or very young children. The data presented here show that our DTS self-collection method can be performed by patients/subjects in isolation, such as in their own home, apartment, or car (such as in drive-thru testing), without the need for PPE. A limitation of our self-collection is that this method does not distinguish between sputum and saliva, which is not expected to alter the test efficiency. Other protocols for DTS also contain sputum and saliva, and have better accuracy when requiring patients to cough, which is required in our study (Won et al., 2020). To collect solely sputum samples would be resource intensive as lavage would be needed and thus, would not be practical.

The other advantage of the PSCS-CoV2 test method is the viral inactivating solution (VIS). In addition to inactivating the virus, VIS helps stabilize the RNA. We demonstrate that RNA in VIS are stable for at least 56 h in temperature ranging from 4 °C to 40 °C. In addition, our limits of detection experiment shows that we can detect at least 1/10^4^ dilution of a DTS sample from a positive patient. In the case of pooled samples, putting multiple samples together could end up diluting a positive sample. The LoD data indicate that numerous samples can be pooled without loss of sensitivity. In addition, we demonstrated by serial collection of samples from an individual patient over a ten-day period that the method is sensitive from the peak of infection to late in the course of the infection, when the amount of virus present in the patient (and sample) is greatly reduced.

Our collection and testing protocol shows 100% sensitivity (95% CI [92–100%]) and 100% specificity [95% CI, 89–100] for SARS-CoV-2. A limitation is that all the positive patients were symptomatic for COVID-19, but not severe enough to need hospitalization. The severity of COVID-19 affects the accuracy of testing for SARS-CoV-2 (Won et al., 2020). Another limitation of these metrics is the limited number of samples. In addition, a limitation is that we compare our *PSCS-CoV2* test to tests that involve nasopharyngeal swab collection. While nasopharyngeal tests are ubiquitously used and have FDA approval, a better gold standard would be bronchoalveolar lavage fluid or double nasopharyngeal swabs since the sensitivity for SARS-CoV-2 is better than nasopharyngeal swabs (Won et al., 2020). The strong diagnostic metrics for our protocol is due to the high threshold for a sample to be positive. We required a cycle threshold limit for 2 SARS-CoV-2 markers. In addition, we required a cycle threshold (Ct) limit for RP, which served as a positive control for the integrity of the RNA sample. We also utilized viral inactivation and stability solution, which helps preserve RNA, allowing for higher quality samples. We utilized a very high cycle threshold of 40 to be the cutoff between a positive and negative result to ensure that we have a low false negative rate. This Ct value is higher than the Ct used by some other laboratories (Engelmann et al., 2021). Many labs utilize different Ct cutoffs, and do not make their Ct values public. Understandably, there are multiple reasons the Ct values can vary from lab to lab, including transport, time from infection to collection to analysis, and method of specimen collection.

We calculated optimal pooling size based on positivity rate, along with showing the pooling strategy in practice. Pooling of four samples and pooling of two samples worked out well. For pools of four, we ended up running half the number of tests with this method than if we individually tested each sample. For pools of two, we ended up running 2/3 the number of trials with the pooling method rather than if we individually tested each sample. Our positivity rate was below 14% for samples in Pool A and samples in Pool B.

An issue with the pooling method was that we did not know the positivity rate beforehand. We can make an approximation of the positivity rate based on the government reported positivity rate in the state or county where the samples were collected. We can also factor if the samples are at high risk for having SARS-CoV2 based on symptoms or recent exposure to COVID-19 patients.

The pooling method is not optimal for positivity rate greater than 0.29. This is in line with previous research that notes pooling is not worth it for prevalence of positive samples greater than 30% (Williams, 2010). We suggest that the pooling method be utilized when the positivity rate was half that of our recommended positivity rate for k (pool size). This was because running the samples at two different times was approximately equivalent to twice the amount of work, so one should have half the number of expected total trials to run. In addition, our algorithm notes pool sizes of two and four are approximately equivalent. Thus, if the noted positivity rate is less than 0.15, we recommend using the larger pool size of four.

A limitation with the pooling method is that it may increase the false negative rate. A COVID-19 positive patient would need to test positive twice, first in the pool and then individually. In addition, pooling may dilute the concentration of the SARS-CoV2 RNA, reducing the sensitivity of the pooled test. Hence, we loosened the requirements to test a pool for individual sample, only requiring that the cycle threshold < 40 for N1 or < 40 for a second SARS-CoV-2 marker. To count an individual sample positive for COVID-19, the cycle threshold < 40 for both SARS-CoV-2 marker. In addition, our limits of detection studies showed we could dilute our samples at least to a 1/10^4^ dilution and still identify a positive sample.

One limitation of our study is the subjects recruited, who all came from a local college campus. We did not collect demographics data, but it is likely the subjects were primarily young adults who were relatively healthy with minimal underlying health conditions. Further studies with a broader cohort of subjects and COVID-19 disease severity, with a wider age range would provide further validity and applicability to our results. Another limitation is the timing of the collection after a patient is tested positive for SARS-CoV2. Patients underwent self-collection within 72 h of being tested positive, which gives times for the viral load SARS-CoV2 to decrease. In addition, with the rise of the Omnicron variant, further studies are needed to test the performance of our assay on the Omnicron variant. While the Omnicron variant has a mutation in the N1 gene, a pre-print has shown that the mutation does not affect CDC N1 target detection (Bei et al., 2021).

## Conclusions

Testing for the SARS-CoV-2 virus remains an essential tool to ensure the health of the world population as the virus continues to mutate and as regions return to normalcy. Our goal was to develop and evaluate an affordable and convenient testing method for COVID-19 that, through effective distribution, could be utilized in widespread efforts. Through the creation of our Patient Self-Collection of Sample-CoV2 (*PSCS-CoV2*) method, we were able to demonstrate the viability of our collection and extraction protocols, with 100% sensitivity and specificity. To further conserve resources and reduce costs, we sought out an effective pooling strategy based on local estimated positivity rates, showing a proof of concept for a pool size of 4. Further research is needed for more accurate sensitivity and specificity of our *PSCS-CoV2* method. Therefore, through the combined efforts of our *PSCS-CoV2* method and pooling strategy, we could implement a simple and cost-effective testing system in regions short on medical resources across the world.

##  Supplemental Information

10.7717/peerj.13277/supp-1 Supplemental Information 1 Picture of the Two-Step Kit container In the picture is a sample collection tube (a 50 mL self-standing centrifuge tube with an orange cap) and a smaller tube (5 mL self-standing tube with a white cap). The smaller tube contains a virus-inactivating and RNase-inhibiting solution. The sample collection tube is in a sealable biohazard plastic bag.

10.7717/peerj.13277/supp-2 Supplemental Information 2 Cycle threshold of serial saliva samples collected over time from a positive COVID-19 individual RNA isolated from saliva collected *via* the PSCS-CoV2 method. RNA was amplified for RP, N1, and N2. Day 0 was the first collection from a confirmed positive COVID-19 individual. Saliva samples were serially collected at day 3, 4, 5, 6, 7, 8, 9, 10 and 11. Cycle threshold for N1 and N2 generally increased over time while the cycle threshold for RP stayed relatively constant.

10.7717/peerj.13277/supp-3 Supplemental Information 3 Accuracy of the PSCS-CoV2 for detection of SARS-CoV2 positive and negative samples n.d = not detected, cycle threshold growth curves that do not cross the baseline before 40 cycles. NP = Nasopharyngeal swab. Ct = cycle threshold. PSCS-CoV2 = method of Patient Self-Collection of Sample for SARS-CoV-2. Ct for the RP of all the samples (positive cases and negative cases) were less than 34.

10.7717/peerj.13277/supp-4 Supplemental Information 4 Raw data for all the tables and supplementary figures

## Additional Information and Declarations

### Data Availability

The following information was supplied regarding data availability: The raw data is available in the Supplementary File.
