# Supplementary material for: An open source and convenient method for the wide-spread testing of COVID-19 using deep throat sputum samples
Source: PeerJ. 2022 May 10;10:e13277. doi: 10.7717/peerj.13277 (PMC9104087; doi:10.7717/peerj.13277)
Supplement: Supplemental Information 1 — In the picture is a sample collection tube (a 50 mL self-standing centrifuge tube with an orange cap) and a smaller tube (5 mL self-standing tube with a white cap). The smaller tube contains a virus-inactivating and RNase-inhibiting solution. The sample collection tube is in a sealable biohazard plastic bag. [file peerj-10-13277-s001.pdf]

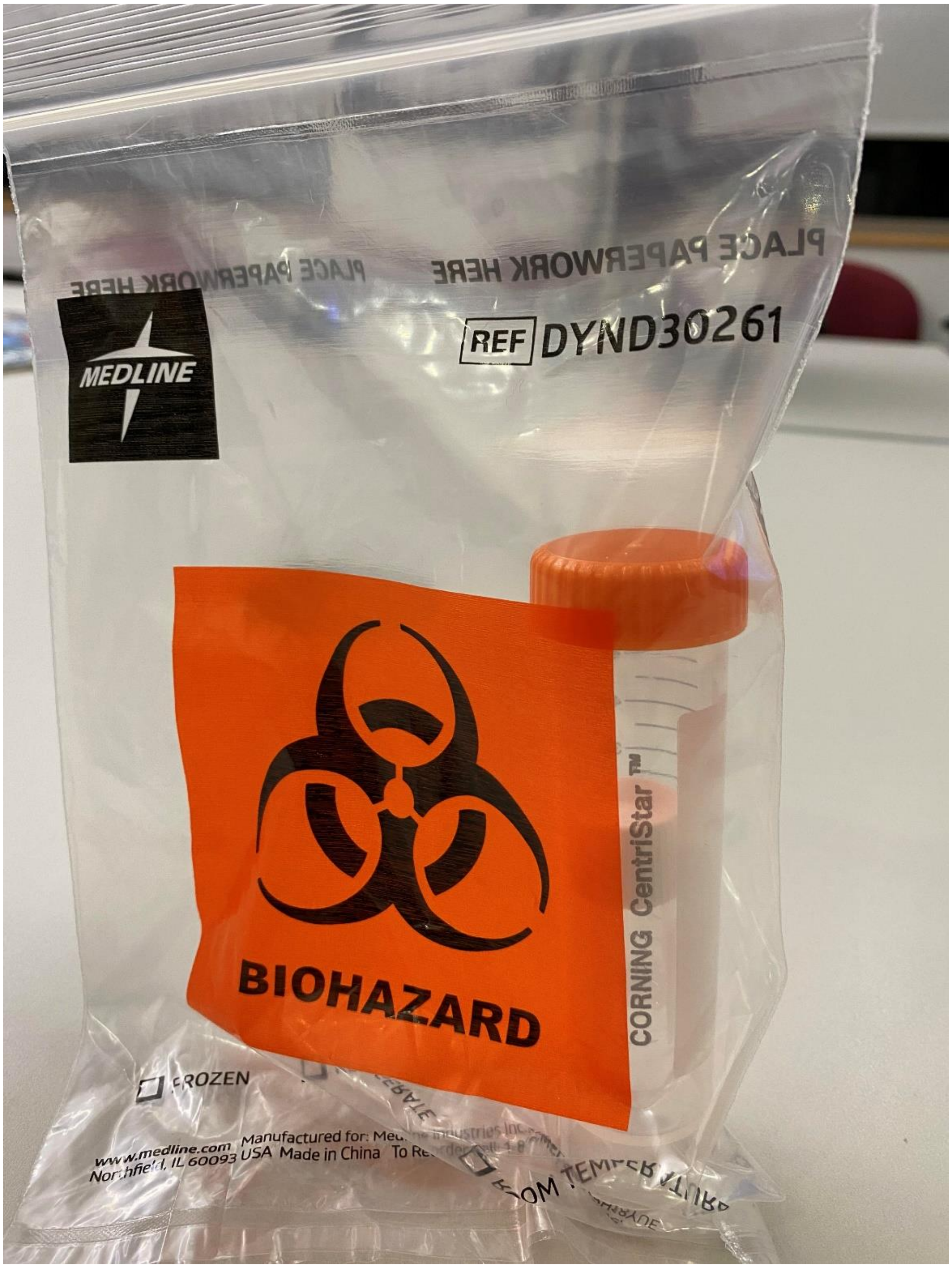

Supplemental Figure 1: Picture of the Two-Step Kit container. In the picture is a sample collection tube (a 50 mL self-standing centrifuge tube with an orange cap) and a smaller tube (5 mL self-standing tube with a white cap). The smaller tube contains a virus-inactivating and RNase-inhibiting solution. The sample collection tube is in a sealable biohazard plastic bag.
