# Supplementary material for: An open source and convenient method for the wide-spread testing of COVID-19 using deep throat sputum samples
Source: PeerJ. 2022 May 10;10:e13277. doi: 10.7717/peerj.13277 (PMC9104087; doi:10.7717/peerj.13277)
Supplement: Supplemental Information 2 — RNA isolated from saliva collected via the PSCS-CoV2 method. RNA was amplified for RP, N1, and N2. Day 0 was the first collection from a confirmed positive COVID-19 individual. Saliva samples were serially collected at day 3, 4, 5, 6, 7, 8, 9, 10 and 11. Cycle threshold for N1 and N2 generally increased over time while the cycle threshold for RP stayed relatively constant. [file peerj-10-13277-s002.pdf]

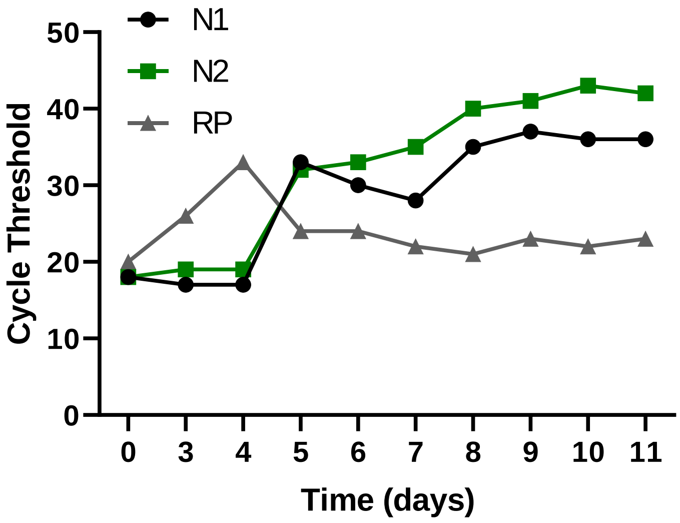

Supplemental Figure 2: Cycle threshold of serial saliva samples collected over time from a positive COVID-19 individual.

RNA isolated from saliva collected via the PSCS-CoV2 method. RNA was amplified for RP, N1, and N2. Day 0 was the first collection from a confirmed positive COVID-19 individual. Saliva samples were serially collected at day 3, 4, 5, 6, 7, 8, 9, 10 and 11. Cycle threshold for N1 and N2 generally increased over time while the cycle threshold for RP stayed relatively constant.
