# Supplementary material for: An open source and convenient method for the wide-spread testing of COVID-19 using deep throat sputum samples
Source: PeerJ. 2022 May 10;10:e13277. doi: 10.7717/peerj.13277 (PMC9104087; doi:10.7717/peerj.13277)
Supplement: Supplemental Information 3 — n.d = not detected, cycle threshold growth curves that do not cross the baseline before 40 cycles. NP = Nasopharyngeal swab. Ct = cycle threshold. PSCS-CoV2 = method of Patient Self-Collection of Sample for SARS-CoV-2. Ct for the RP of all the samples (positive cases and negative cases) were less than 34. [file peerj-10-13277-s003.docx]

**Supplemental Table 1:**

**Accuracy of the PSCS-CoV2 for detection of SARS-CoV2 positive and negative samples.**

| **Patient Type (total cases)** | **Sample Type** | **Analysis** | **Target** | | |
| --- | --- | --- | --- | --- | --- |
|  |  |  | **N1** | **N2** | **E** |
| **Positive Cases (42)** | **NP Swab** | **Positives/Total** | **42/42** | **42/42** | **not tested** |
|  |  | **Ct** | **<40** | **<40** | **not tested** |
|  | **PSCS-CoV2**  **Sputum** | **Positives/Total** | **42/42** | **not tested** | **42/42** |
|  |  | **Ct** | **<40** | **not tested** | **<40** |
| **Negative Cases (30)** | **NP Swab** | **Positives/Total** | **0/30** | **0/30** | **not tested** |
|  |  | **Ct** | **n.d.** | **n.d.** | **not tested** |
|  | **PSCS-CoV2**  **Sputum** | **Positives/Total** | **0/30** | **0/30** | **0/30** |
|  |  | **Ct** | **n.d.** | **n.d.** | **n.d.** |

n.d=not detected, cycle threshold growth curves that do not cross the baseline before 40 cycles. NP = Nasopharyngeal swab. Ct = cycle threshold. PSCS-CoV2 = method of Patient Self-Collection of Sample for SARS-CoV-2. Ct for the RP of all the samples (positive cases and negative cases) were less than 34.
